# Inhibition of CHK1 enhances cell death induced by the Bcl-2-selective inhibitor ABT-199 in acute myeloid leukemia cells

**DOI:** 10.18632/oncotarget.9185

**Published:** 2016-05-05

**Authors:** Jianyun Zhao, Xiaojia Niu, Xinyu Li, Holly Edwards, Guan Wang, Yue Wang, Jeffrey W Taub, Hai Lin, Yubin Ge

**Affiliations:** ^1^ National Engineering Laboratory for AIDS Vaccine, Key Laboratory for Molecular Enzymology and Engineering, Ministry of Education, School of Life Sciences, Jilin University, Changchun, China; ^2^ Department of Oncology, Wayne State University School of Medicine, Detroit, MI, USA; ^3^ Molecular Therapeutics Program, Barbara Ann Karmanos Cancer Institute, Wayne State University School of Medicine, Detroit, MI, USA; ^4^ Department of Pediatric Hematology and Oncology, The First Hospital of Jilin University, Changchun, China; ^5^ Department of Pediatrics, Wayne State University School of Medicine, Detroit, MI, USA; ^6^ Division of Pediatric Hematology/Oncology, Children's Hospital of Michigan, Detroit, MI, USA; ^7^ Department of Hematology and Oncology, The First Hospital of Jilin University, Changchun, China

**Keywords:** LY2603618, ABT-199, acute myeloid leukemia, CHK1, Mcl-1

## Abstract

Resistance to standard chemotherapy agents remains a major obstacle for improving treatment outcomes for acute myeloid leukemia (AML). The Bcl-2-selective inhibitor ABT-199 has demonstrated encouraging preclinical results, drug resistance remains a concern. Mcl-1 has been demonstrated to contribute to ABT-199 resistance, thus combining with therapies that target Mcl-1 could overcome such resistance. In this study, we utilized a CHK1 inhibitor, LY2603618, to decrease Mcl-1 and enhance ABT-199 efficacy. We found that LY2603618 treatment resulted in abolishment of the G2/M cell cycle checkpoint and increased DNA damage, which was partially dependent on CDK activity. LY2603618 treatment resulted in decrease of Mcl-1, which coincided with the initiation of apoptosis. Overexpression of Mcl-1 in AML cells significantly attenuated apoptosis induced by LY2603618, confirming the critical role of Mcl-1 in apoptosis induced by the agent. Simultaneous treatment with LY2603618 and ABT-199 resulted in synergistic induction of apoptosis in both AML cell lines and primary patient samples. Our findings provide new insights into overcoming a mechanism of intrinsic ABT-199 resistance in AML cells and support the clinical development of combined ABT-199 and CHK1 inhibition.

## INTRODUCTION

Acute myeloid leukemia (AML) remains a devastating disease. The overall survival rate for adults is only about 25% [1] and although the overall survival rate for children is around 65% [2], it lags significantly behind that of acute lymphoblastic leukemia, which is about 90% for the pediatric population [3]. A major cause of treatment failure is resistance to cytarabine (ara-C) and anthracycline [e.g., daunorubicin (DNR)]-based chemotherapy [4]. Thus, novel therapies are needed to overcome chemoresistance and improve the overall survival of AML patients.

One strategy to overcome chemoresistance is to target the anti-apoptotic Bcl-2 proteins, as they have been demonstrated to be associated with poor clinical outcome and chemoresistance in leukemic cell line models [5–8]. ABT-199 is the first Bcl-2-selective inhibitor that has demonstrated promising results in multiple cancers, including AML [9–15], though drug resistance remains a concern. We and others have previously demonstrated that Mcl-1 plays a key role in resistance to ABT-199 [12, 16–18]. In our most recent study, we demonstrate that induction of Mcl-1 by ABT-199 represents an intrinsic mechanism of resistance to the agent in AML cells [19]. Thus, rationally designed combinations could prove to be a more effective option for the treatment of AML with ABT-199.

Targeting the DNA damage response (DDR) is another approach to overcome chemotherapy resistance. The DDR involves multiple signaling pathways through which cells maintain genomic integrity following various stresses [20–23]. Checkpoint kinase 1 (CHK1) plays a central role in the DDR and its inhibition can affect replication initiation, replication fork stability, homologous recombination repair, progression of the cell cycle, and the S and G2/M cell cycle checkpoints [20–22, 24]. In addition, CHK1 inhibition has been demonstrated to result in DNA damage [25] and DNA damage has been shown to result in decrease of Mcl-1 expression [26]. Therefore, we hypothesize that inhibition of CHK1 may enhance the cytotoxic effects of ABT-199 by decreasing Mcl-1 expression.

Here, we evaluated the CHK1-selective inhibitor LY2603618 in combination with ABT-199 in AML cell lines and primary patient samples. We demonstrated that LY2603618 treatment resulted in DNA damage and decrease of Mcl-1 expression, which coincided with the initiation of apoptosis. Simultaneous combination of LY2603618 and ABT-199 resulted in synergistic induction of cell death in both AML cell lines and primary patient samples. These findings provide new insights into overcoming intrinsic ABT-199 resistance in AML cells and supports clinical development of the combination of ABT-199 and CHK1 inhibitors.

## RESULTS

### LY2603618 has antileukemic activity against primary AML patient samples and AML cell lines

**Figure 1:**
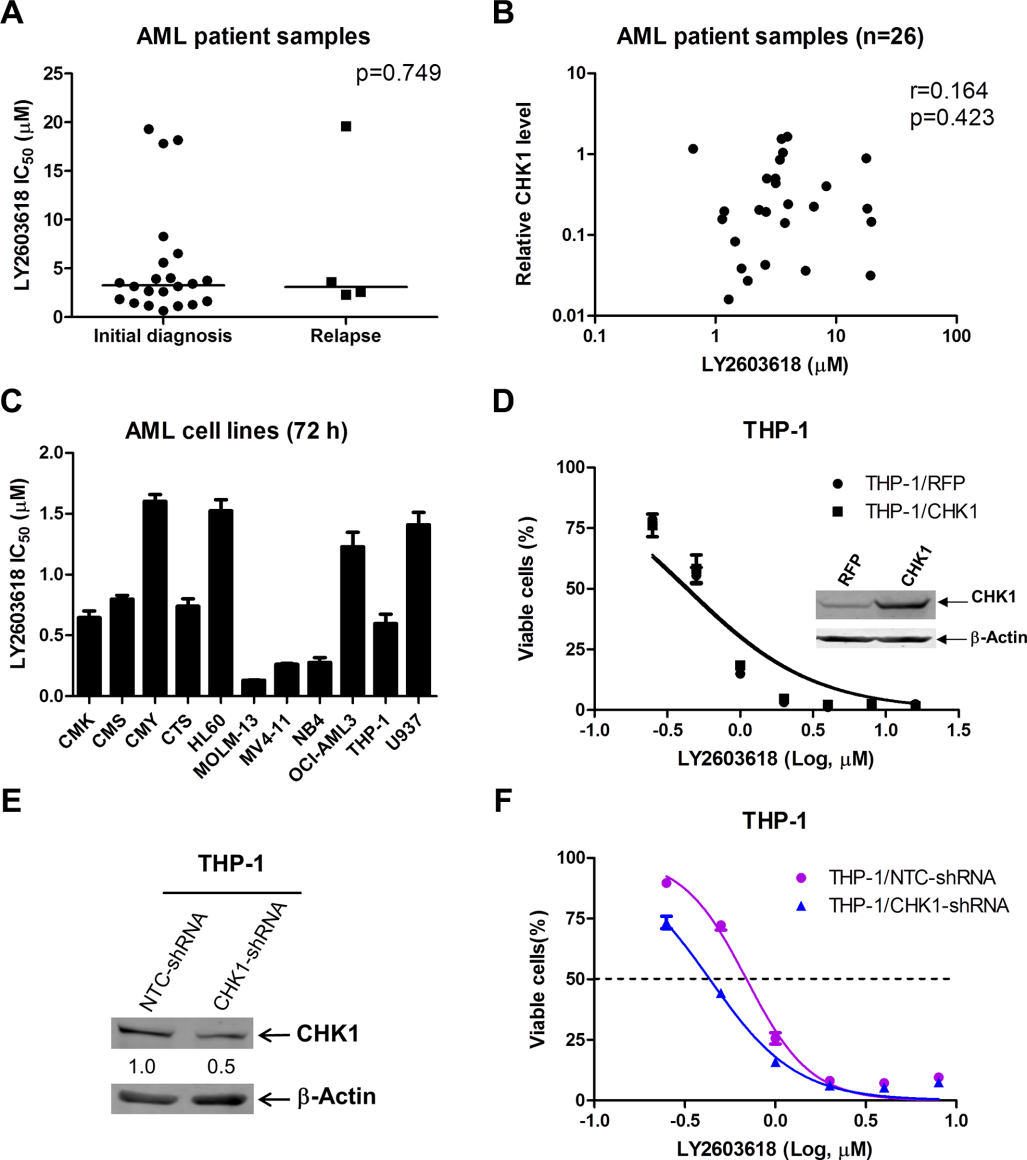
AML cells are sensitive to LY2603618 (**A**) Primary AML patient samples were treated with variable concentrations of LY2603618 in 96-well plates for 72 h and viable cells were determined using MTT reagent. IC_50_ values were calculated as drug concentration necessary to inhibit 50% OD_590_ compared to vehicle control treated cells. The IC_50_ values are means of duplicates from one experiment due to limited sample. The horizontal lines indicate the median. (**B**) Total RNAs were isolated from primary AML patient samples and gene transcript levels were determined by Real-time RT-PCR. Transcript levels were normalized to GAPDH and relative expression levels were calculated using the comparative *Ct* method. The relationship between the CHK1 expression levels and LY2603618 sensitivities was determined by the nonparametric Spearman rank correlation coefficient. (**C**) LY2603618 sensitivities for AML cell lines were determined using MTT assays. (**D**) THP-1 cells were infected with Precision LentiORF CHK1 (THP-1/CHK1) or red fluorescent protein control (THP-1/RFP) lentivirus. LY2603618 sensitivities were determined using MTT assays. Whole cell lysates were subjected to Western blotting (Western blot from a previous publication [28]). (**E** and **F**) THP-1 cells were transiently infected with CHK1- or non-target control (NTC)-shRNA lentivirus particles overnight, the cells were washed and resuspended in fresh complete media and cultured for 48 h. Then Western blotting and MTT assays were perform in the cells to determine CHK1 knockdown (E) and sensitivity to LY2603618 (F).

To investigate LY2603618 sensitivity in AML patient samples and cell lines, first we determined *ex vivo* LY2603618 sensitivity in freshly isolated primary AML blast samples obtained either at initial diagnosis (*n* = 22) or at relapse (*n* = 4) by MTT (3-[4, 5-dimethyl-thiazol-2-yl]-2, 5-diphenyltetrazoliumbromide) assays. For the majority of the primary patient samples (*n* = 23), the LY2603618 IC_50_s were less than 9 μM, which is the maximum clinically achievable concentration of LY2603618 [27]. Interestingly, there was no significant difference between the median LY2603618 IC_50_ for the AML blasts obtained at initial diagnosis and relapse (*p* = 0.749, Table 1 and Figure 1A). Next, we determined CHK1 transcript levels in the primary AML patient samples by real-time RT-PCR. CHK1 transcript levels did not correlate with LY2603618 sensitivities in the samples (Figure 1B). Then we tested LY2603618 sensitivities in 11 AML cell lines by MTT assays. The IC_50_s of LY2603618 in these cell lines ranged from about 0.1–1.6 μM after 72 h treatment (Figure 1C). Consistent with the lack of a correlation between CHK1 transcript levels and LY2603618 IC_50_s in the primary patient samples, ectopic expression of CHK1 in THP-1 AML cell line had no impact on LY2603618 sensitivity, as assessed by MTT assay (Figure 1D, the western blot confirming overexpression was published previously [28]). However, shRNA knockdown of CHK1 (50% decrease of CHK1 protein compared to NTC shRNA) resulted in a significant increase of LY2603618 sensitivity in THP-1 cells (1.6-fold, *p* = 0.023, Figure 1E and 1F).

**Table 1:** Patient characteristics and LY2603618 sensitivity in primary AML patient samples (*n* = 26)

Patient	Gender	Age (year)	Disease status	FAB subtype	Cytogenetics	Blast purity (%)	LY2603618 IC50 (μM)	Relative CHK1 transcripts
**AML#8**	Male	55	Newly diagnosed	M2	46, XY	31.5	5.6	0.04
**AML#9**	Female	35	Relapsed	M5	47, XX,+10,t(16;21)(p11;q22),add(11p)	87	2.6	0.04
**AML#10**	Male	23	Newly diagnosed	M2	46, XY, del(9q)	52.5	1.2	0.20
**AML#11**	Female	43	Newly diagnosed	M2	46, XX	91	1.8	0.03
**AML#12**	Male	6	Newly diagnosed	M3	46, XY, t (15;17) (q22;q21)	85	3.8	0.14
**AML#15**	Female	63	Newly diagnosed	M2	46, XX	23	1.6	0.04
**AML#16**	Male	38	Newly diagnosed	M3	46, XY, t (15;17) (q22;q21)	90	1.3	0.02
**AML#17**	Female	45	Newly diagnosed	M4	46, XX	35.5	18.2	0.21
**AML#18**	Female	19	Relapsed	M2	46, XX, t (8;21) (q22;q22)/46, XX	54	2.3	0.20
**AML#19**	Male	4	Newly diagnosed	M4	46, XY	40.5	8.3	0.40
**AML#21**	Male	42	Newly diagnosed	M4	46, XY/44, XY, −17, −19, (11q-?)	51	3.2	0.44
**AML#23**	Male	60	Newly diagnosed	M5	48, XY,+2,+8, i (12) (q10)/46, XY	81	3.9	1.65
**AML#24**	Male	59	Newly diagnosed	M2	46, XY	42	19.3	0.03
**AML#25**	Female	12	Newly diagnosed	M3	46, XX, t (15;17) (q22;q21)/46, XX	88	1.1	0.16
**AML#27**	female	41	Relapsed	M4	47, XX, del (5q), +8, t (15;18) (q12,q23)	24	19.6	0.15
**AML#29**	Male	9	Newly diagnosed	M4	46, XY, t (6; 9) (p22;q34)/46, XY	31	1.5	0.08
**AML#31**	Male	17	Newly diagnosed	M2	46, XY	68.5	0.7	1.17
**AML#33**	Female	76	Newly diagnosed	M5	46, XX	84.5	3.5	1.55
**AML#35**	Male	65	Newly diagnosed	M5	47, XY, add (7q), −16, −17, +marx3	76	17.8	0.89
**AML#36**	Male	43	Newly diagnosed	M2	46, XY, t (8;21) (q22;q22)	48	2.7	0.50
**AML#39**	Male	50	Newly diagnosed	M2	45, X, -Y, t (8;21) (q22;q22), del (11q)	46	4.0	0.24
**AML#40**	Male	12	Newly diagnosed	M3	46, XY, t (15;17) (q22;q21)	92.5	2.6	0.19
**AML#41**	Male	74	Newly diagnosed	M5	47, XY,+8	95	6.5	0.23
**AML#43**	Male	19	Newly diagnosed	M2	45, X, -Y, t (8;21) (q22;q22), del (9q)	47	3.4	0.85
**AML#44**	Male	25	Newly diagnosed	M3	46, XY, t (15;17) (q22;q21)	94	3.2	0.50
**AML#45**	Male	48	Relapsed	M2	46, XY, t (7;11) (p15;p15)	39.5	3.6	1.04

### LY2603618 induces bak-dependent apoptosis in AML cell lines

**Figure 2:**
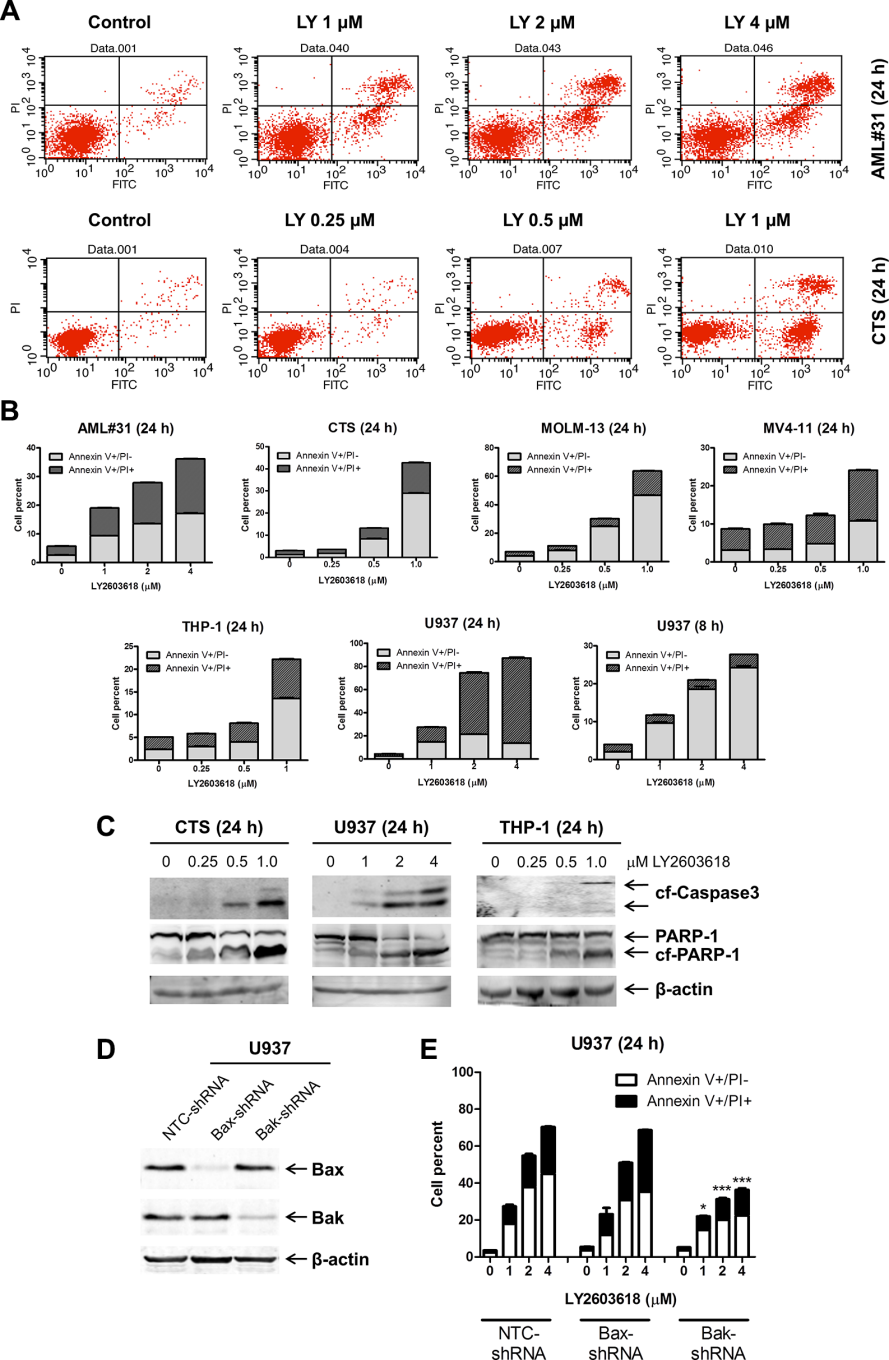
LY2603618 induces apoptosis in AML cell lines and a primary patient sample (**A**) AML cells were treated with LY2603618 (LY) for 24 h and then subjected to Annexin V-FITC/PI staining and flow cytometry analyses. Representative dot plots are shown for primary patient AML#31 (top) and CTS cells (bottom). (**B**) Mean percent Annexin V+ cells ± s.e.m are shown. (**C**) Whole cell lysates were subjected to Western blotting. (**D**) U937 cells were infected with NTC-, Bax- or Bak-shRNA lentivirus particles overnight, then washed and incubated for 48 h prior to adding puromycin to the culture medium. Whole cell lysates were subjected to Western blotting. (**E**) U937 NTC-, Bax-, and Bak-shRNA cells were treated with LY for 24 h and then subjected to Annexin V/PI staining and flow cytometry analysis. *indicates *p* < 0.05, ***indicates *p* < 0.0005.

To assess the effect of LY2603618 treatment on cell death, first we treated five AML cell lines (CTS, MOLM-13, MV4-11, THP-1, and U937) and one primary AML patient sample (AML #31) with variable concentrations of LY2603618 for 24 h and then subjected them to Annexin V/propidium iodide (PI) staining, and flow cytometry analyses. LY2603618 treatment resulted in concentration-dependent increase in Annexin V positive cells (Figure 2A and 2B). The U937 cells treated with LY2603618 for 24 h were mostly dead, as indicated by AnnexinV+/PI+, so the treatment was performed with a shorter incubation to determine if the cells underwent apoptosis. After 8 h treatment, U937 cells showed concentration-dependent increase in AnnexinV+PI- cells, indicating that the cells underwent apoptosis. LY2603618-induced cell death, in all cell lines tested, was accompanied by cleavage of caspase 3 and PARP (poly ADP ribose polymerase, Figure 2C), demonstrating that the cells underwent apoptosis. To determine if the cells did indeed undergo intrinsic apoptosis, we performed shRNA knockdown of Bax and Bak, at least one of which is required for intrinsic apoptosis [29]. The LY2603618-induced increase of Annexin V positive cells was diminished by shRNA knockdown of Bak, but not Bax, demonstrating that LY2603618 induces Bak-dependent intrinsic apoptosis in AML cells (Figure 2D and 2E).

### LY2603618 treatment decreases the G2/M cell population and increases DNA damage in AML cell lines

**Figure 3:**
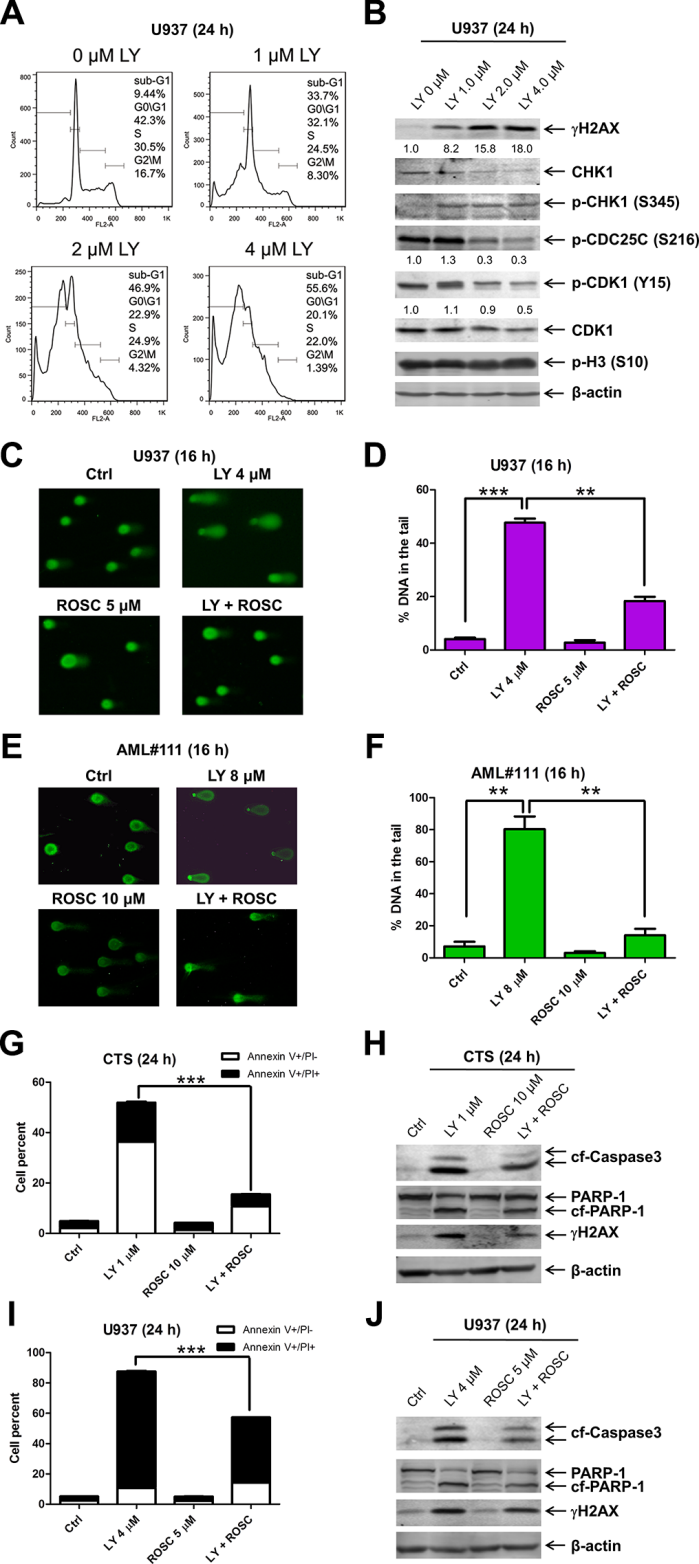
LY2603618 treatment results in DNA double strand breaks (**A**) U937 cells were treated with LY for 24 h, then fixed with ethanol and stained with PI for cell cycle analysis. (**B**) U937 cells were treated with LY for 24 h. Whole cell lysates were subjected to Western blotting. The fold changes for the γH2AX, p-CDC25C, and p-CDK1 densitometry measurements, normalized to β-actin and then compared to no drug treatment control, are indicated. (**C**) U937 cells were treated with LY and Roscovitine (ROSC), alone or in combination, for 16 h and then subjected to alkaline comet assay analysis. Representative images are shown. (**D**) Comet assay results are graphed as median percent DNA in the tail from 4 replicate gels ± s.e.m. **indicates *p* < 0.005 and ***indicates *p* < 0.0005. (**E**) Primary AML patient sample AML#111 was treated with LY and Roscovitine (ROSC), alone or in combination, for 16 h and then subjected to alkaline comet assay analysis. Representative images are shown. (**F**) Comet assay results are graphed as median percent DNA in the tail from 4 replicate gels ± s.e.m. **indicates *p* < 0.005. (**G**–**J**) CTS and U937 cells were treated with LY and ROSC, alone or in combination for 24 h. The cells were subjected to Annexin V/PI staining and flow cytometry analysis (G and I). ***indicates *p* < 0.0005. Whole cell lysates were subjected to Western blotting (H&J).

To investigate the effects of LY2603618 treatment on cell cycle progression, we treated CTS, THP-1, and U937 cells with LY2603618 for 24 h. PI staining and flow cytometry analyses revealed a concentration-dependent decrease of the G2/M population accompanied by concentration-dependent increase of sub-G1 population (Figures 3A, S1 and S2). Decreased protein levels for CHK1 were detected after LY2603618 treatment of the U937 cells. Although treatment with high concentrations of LY2603618 resulted in decreased levels of p-CDC25C and p-CDK1 (phosphorylated cyclin-dependent kinase 1), it did not alter the levels of phosphorylated histone H3 (p-H3, a marker for mitosis) (Figure 3B). Increased levels of p-CHK1 (S345), indicative of DNA damage, were detected in LY2603618 treated cells. In addition, there was a concentration-dependent increase of γH2AX post LY2603618 treatments, further suggesting that LY2603618 induced DNA damage in the cells.

To evaluate if LY2603618 treatment truly induced DNA damage and determine the relationship between the induced DNA damage and CDK activity, U937 cells were treated with LY2603618 and Roscovitine (a CDK inhibitor), alone and in combination, for 16 h and then subjected to the alkaline comet assay. As shown in Figure 3C and 3D, LY2603618 treatment resulted in significantly increased percent DNA in the tail, which was significantly attenuated by the addition of Roscovitine. Essentially the same results were also obtained with a primary AML patient sample AML#111 (AML M2, Figure 3E and 3F). In addition, Roscovitine significantly mitigated LY2603618-induced apoptosis in both CTS and U937 cells (Figure 3G and 3I), accompanied by decreased levels of γH2AX, cleaved PARP, and cleaved caspase-3 (Figure 3H and 3J). These results demonstrate that LY2603618 induces DNA damage and apoptosis in AML cells by a process which is at least partially dependent on CDK activity.

### Decrease of Mcl-1 plays an important role in LY2603618-induced apoptosis in AML cell lines

**Figure 4:**
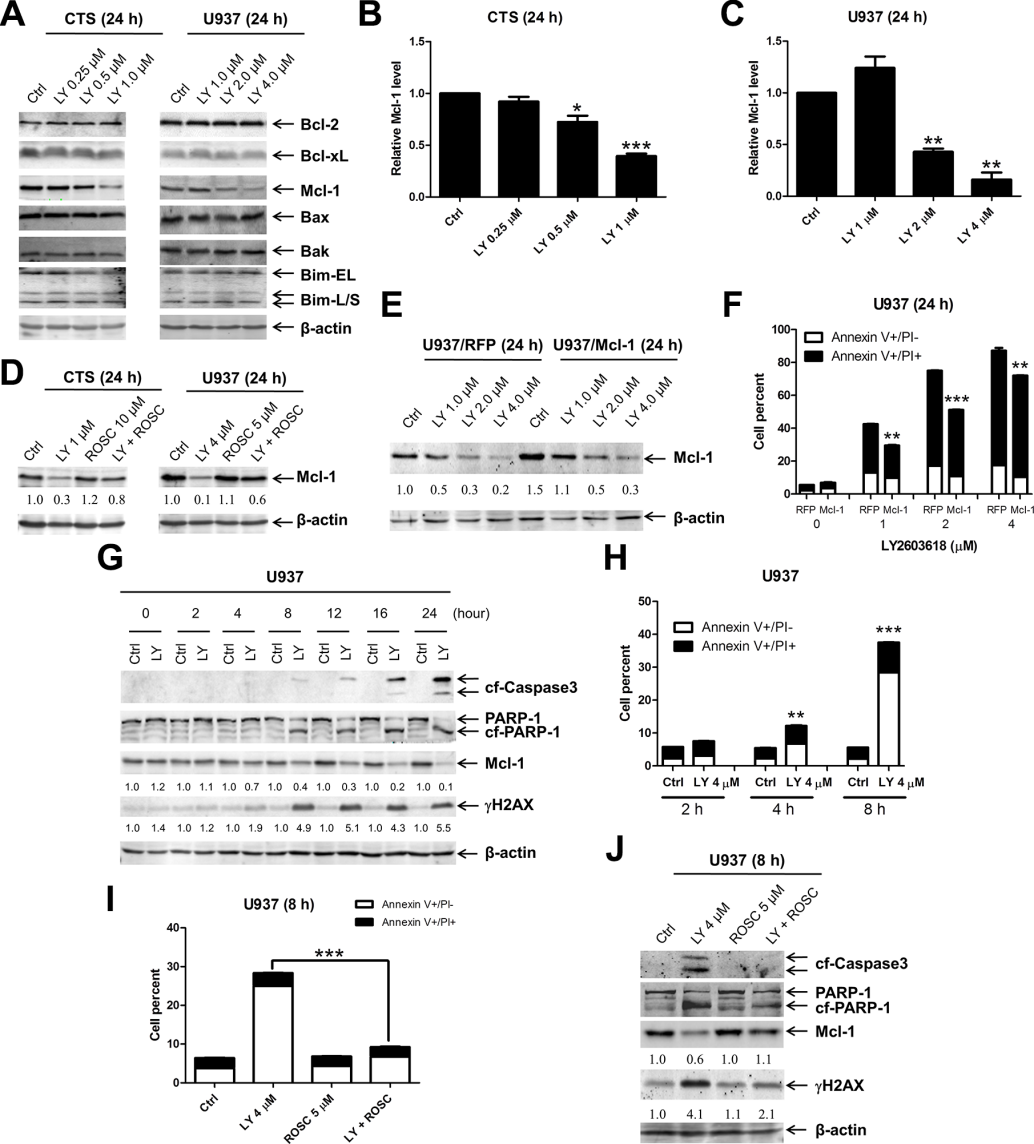
LY2603618 treatment results in decreased expression of Mcl-1 (**A**) CTS and U937 cells were treated with LY for 24 h. Whole cell lysates were subjected to Western blotting. (**B** and **C**) Relative densitometry of Mcl-1 expression were measured using Odyssey Software V3.0 and graphed as fold change compared to the no drug control ± s.e.m. *indicates *p* < 0.05, **indicates *p* < 0.005, and ***indicates *p* < 0.0005. (**D**) CTS and U937 cells were treated with LY and ROSC alone or in combination for 24 h. Whole cell lysates were subjected to Western blotting. The fold changes for the Mcl-1 densitometry measurements, normalized to β-actin and then compared to no drug treatment control, are indicated. (**E**) U937 cells were infected with Precision LentiORF Mcl-1 (U937/Mcl-1) and RFP control (U937/RFP) lentivirus particles overnight, then washed and incubated for 48 h prior to adding puromycin to the culture medium. The puromycin-resistant cells were treated with LY for 24 h. Whole cell lysates were subjected to Western blotting. The fold changes for the Mcl-1 densitometry measurements, normalized to β-actin and then compared to no drug treatment control, are indicated. (**F**) U937/RFP and U937/Mcl-1 cells were treated with LY for 24 h and then subjected to Annexin V/PI staining and flow cytometry analysis. **indicates *p* < 0.005 and ***indicates *p* < 0.0005. (**G**) U937 cells were treated with 4 μM LY for the indicated times. Whole cell lysates were subjected to Western blotting. Mcl-1 densitometry measurements, normalized to β-actin and then compared to no drug treatment control at the corresponding time point, are indicated. (**H**) U937 cells were treated with LY for 2 h, 4 h, and 8 h and then subjected to Annexin V/PI staining and flow cytometry analysis. **indicates *p* < 0.005 and ***indicates *p* < 0.0005. (**I**) U937 cells were treated with LY and ROSC, alone or in combination, for 8 h. Cells were then subjected to Annexin V/PI staining and flow cytometry analysis. ***indicates *p* < 0.0005. (**J**) Whole cell lysates were subjected to Western blotting. Densitometry measurements, normalized to β-actin and then compared to no drug treatment control, are indicated.

It has been previously demonstrated that DNA damage can cause decreased expression of Mcl-1, which plays an important role in DNA damage-induced apoptosis [26]. It is conceivable that LY2603618 treatment induces DNA damage which causes decreased expression of Mcl-1, leading to apoptosis in AML cells. To test this possibility, we investigated the effects of LY2603618 treatment on the Bcl-2 family proteins in U937 cells. While protein levels for Bcl-2, Bcl-xL, Bak, Bax, and Bim remained unchanged post treatment with LY2603618 for 24 h, protein levels for Mcl-1 decreased significantly in cells treated with higher concentrations of LY2603618 (Figure 4A–4C), which correlated with much higher levels of apoptosis induced by the agent (Figure 2B). Interestingly, Roscovitine partially abolished the decrease of Mcl-1 induced by treatment with LY2603618 (Figure 4D), demonstrating that CDK activity contributed to the decrease of Mcl-1. To determine if Mcl-1 inhibits LY2603618-induced apoptosis, we ectopically overexpressed Mcl-1 (U937/Mcl-1) and red fluorescent protein (U937/RFP) in U937 cells (50% increase compared to U937/RFP cells, Figure 4E). As shown in Figure 4F, apoptosis induced by LY2603618 in the U937/Mcl-1 cells was significantly lower than that in U937/RFP cells, demonstrating that decrease of Mcl-1 contributes to LY2603618-induced apoptosis in AML cells.

To further elucidate the relationship among DNA damage, Mcl-1 levels, and apoptosis, we treated U937 cells with LY2603618 for up to 24 h and then subjected whole cell lysates to western blotting. These experiments revealed a time-dependent increase of γH2AX, cleaved PARP and cleaved caspase3 as well as a time-dependent decrease of Mcl-1 (Figure 4G). It is important to note that obvious changes of γH2AX and Mcl-1 began at 4 h and were accompanied by a minor but statistically significant induction of apoptosis by LY2603618 (Figure 4H). To determine if CDK activity contributed to the induction of DNA damage, decrease of Mcl-1, and apoptosis by LY2603618, we treated U937 cells with LY2603618 and Roscovitine, alone or in combination, for 8 h. Roscovitine treatment abrogated LY2603618-induced apoptosis and rescued Mcl-1 protein (Figure 4I and 4J). However, Roscovitine treatment could only partially inhibit LY2603618-induced γH2AX. Similar results were obtained in CTS cells (Figure S3). These results demonstrate that LY2603618 treatment causes decreased expression of Mcl-1 and induces apoptosis in a partially CDK-dependent fashion in AML cells.

### LY2603618 enhances ABT-199-induced apoptosis in AML cell lines

**Figure 5:**
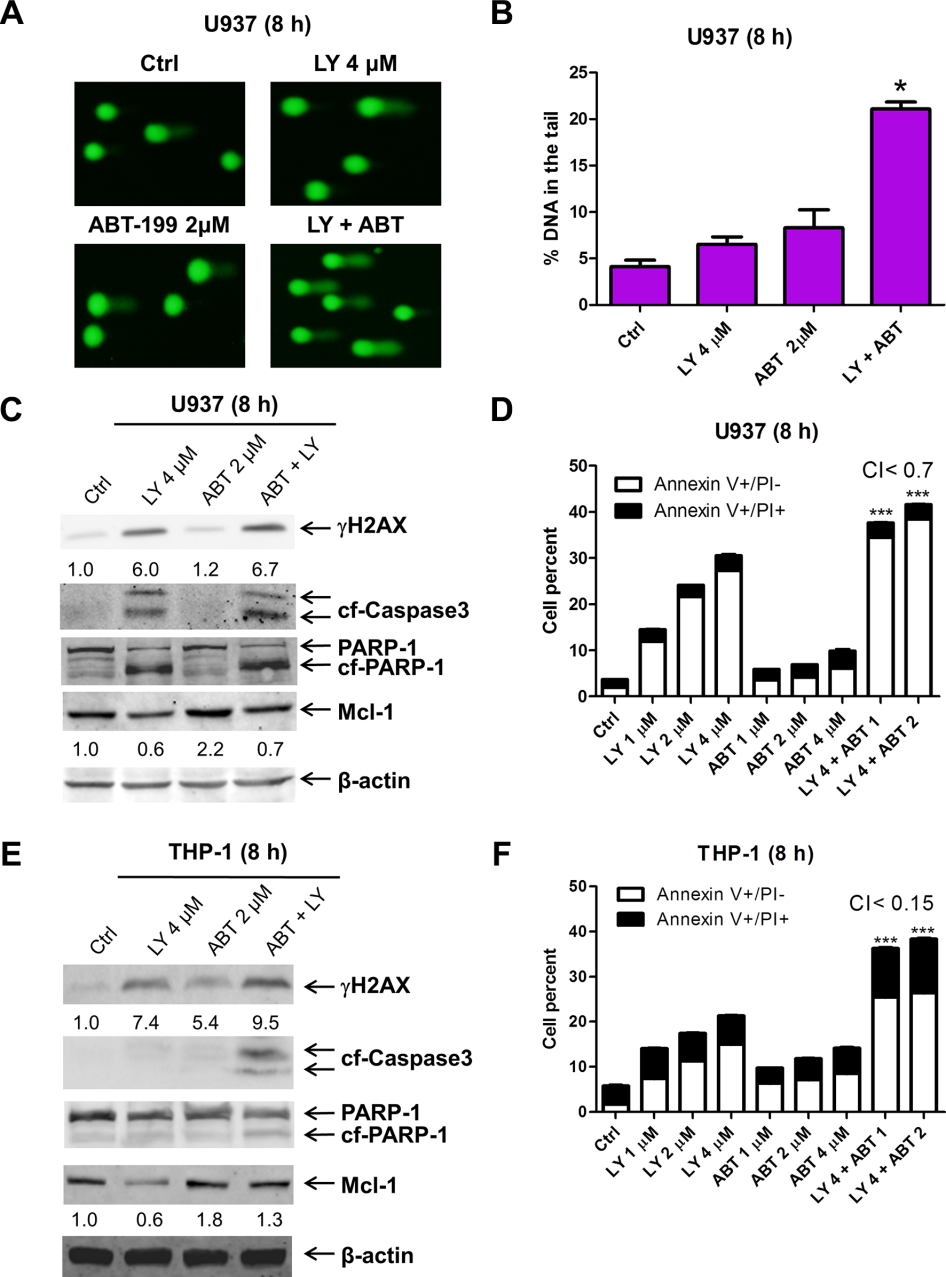
ABT-199 synergizes with LY2603618 in AML cells (**A**) U937 cells were treated with LY and ABT-199 (ABT), alone or in combination, for 8 h and then subjected to alkaline comet assay analysis. Representative images are shown. (**B**) Comet assay results are graphed as median percent DNA in the tail from 3 replicate gels ± s.e.m. * indicates *p* < 0.05. (**C**) Whole cell lysates were subjected to Western blotting. The fold changes for the Mcl-1 densitometry measurements, normalized to β-actin and then compared to no drug treatment control, are indicated. (**D**) U937 cells were treated with LY and ABT-199, alone or in combination, for 8 h and then subjected to Annexin V/PI staining and flow cytometry analysis. ***indicates *p* < 0.0005. (**E**) THP-1 cells were treated with LY and ABT, alone or in combination, for 8 h. Whole cell lysates were subjected to Western blot analysis. (**F**) THP-1 cells were treated with the indicated drugs for 8 h and then subjected to Annexin V/PI staining and flow cytometry analyses. ***indicates *p* < 0.0005.

We and others have demonstrated that Mcl-1 plays an important role in the antileukemic activity of the Bcl-2-selective inhibitor ABT-199 in AML and other blood cancer cells [12, 16–18]. In our most recent study, we demonstrated that induction of Mcl-1 represents an intrinsic mechanism of resistance to ABT-199 in AML cells [19]. Thus, combining LY2603618, which can cause DNA damage and decreased expression of Mcl-1, with ABT-199 may enhance its antileukemic activity in AML cells. To investigate this, we treated the ABT-199 resistant cell line U937 (a relatively resistant AML cell line with an ABT-199 IC_50_ of 13.5 μM, as determined previously [12]) with LY2603618 and ABT-199 for 8 h. LY2603618 or ABT-199 treatment alone did not cause significant DNA damage after 8 h treatment, as assessed by alkaline comet assay (Figure 5A and 5B). However, the combined treatment resulted in a significant increase in DNA damage. In addition, LY2603618 treatment abrogated the increase of Mcl-1 seen with ABT-199 treatment (Figure 5C). Furthermore, the combined drug treatment resulted in synergistic induction of apoptosis in U937 cells (Figure 5D), which was accompanied by increased cleavage of PARP and caspase-3 (Figure 5C). Similar results were also obtained in THP-1 cells (another relatively resistant AML cell line with an ABT-199 IC_50_ of 2.4 μM, as determined previously [12], Figure 5E and 5F).

### LY2603618 enhances ABT-199-induced apoptosis in primary AML patient samples

**Figure 6:**
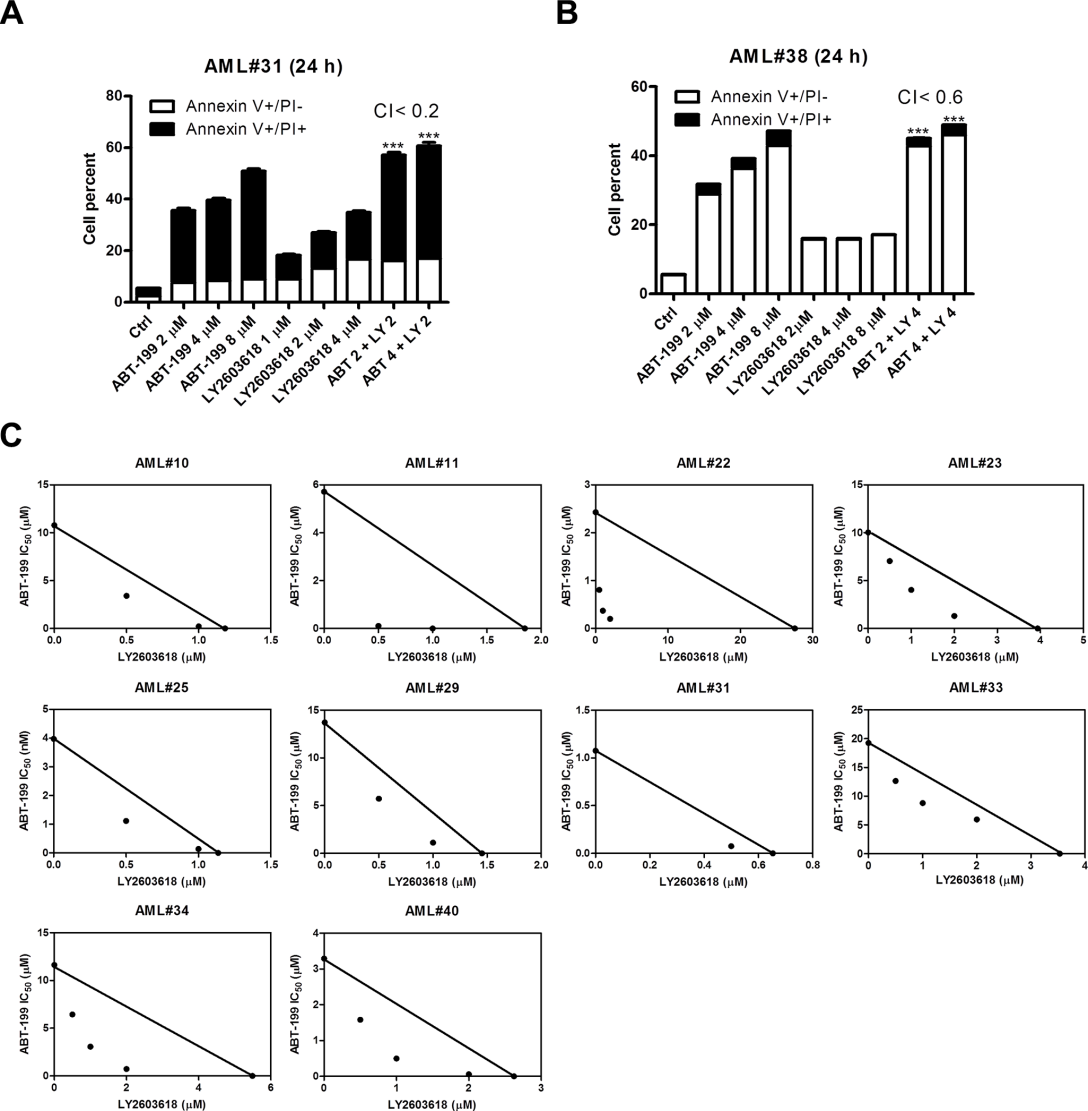
LY2603618 synergizes with ABT-199 in primary AML patient samples (**A** and **B**) Patient samples were treated as indicated for 24 h and then subjected to Annexin V/PI staining and flow cytometry analyses. CI values were calculated using CompuSyn software. ***indicates *p* < 0.0005. (**C**) Primary AML patient samples were treated with ABT-199 and LY for 72 h and then viable cells were determined using MTT reagent. The IC_50_ values are means of duplicates from one experiment due to limited sample. Standard isobologram analysis of antileukemic interactions was performed to determine the extent and direction of the antileukemic interaction. The IC_50_ values of each drug are plotted on the axes; the solid line represents the additive effect, while the points represent the concentrations of each drug resulting in 50% inhibition of proliferation. Points falling below the line indicate synergism whereas those above the line indicate antagonism.

Then we tested the antileukemic effects of LY2603618 and ABT-199 in two primary patient samples which were relatively resistance to ABT-199 (AML#31 ABT-199 IC_50_ was 1 μM and AML#38 ABT-199 IC_50_ was 5 μM, as determined by MTT assays) by Annexin V/PI staining and flow cytometry analyses (patient characteristics are presented in Table 1). In agreement with the AML cell lines, LY2603618 combined with ABT-199 resulted in synergistic induction of cell death (Figure 6A and 6B). Finally, we tested the combined drug treatments in 10 primary AML patient samples which were relatively resistance to ABT-199 (IC_50_s ranging from 1 μM to 20 μM by MTT assays) by MTT assays and standard isobologram analyses. Again, the combined drug treatments resulted in synergistic antileukemic activities against the primary patient samples (Figure 6C).

## DISCUSSION

In this study, we investigated using a CHK1 inhibitor as a DNA damaging agent to decrease Mcl-1 protein levels and enhance ABT-199 sensitivity in AML cells, based on a previous report that DNA damage can cause decreased expression of Mcl-1 in cancer cells [26]. We found that primary patient samples obtained at initial diagnosis and relapse had similar sensitivities to the CHK1 inhibitor LY2603618, suggesting that this agent could be used for treating relapsed AML. We also found that LY2603618 sensitivity did not correlate with CHK1 transcript levels, which is in agreement with Bryant et al. who found that CHK1 protein levels did not correlate with CHK1 inhibitor V158411 sensitivity in leukemia, lymphoma, and lung cancer cell lines [30]. Therefore, CHK1 expression levels probably are not biomarkers for predicting anti-tumor activities of CHK1 inhibitors. Although ectopic overexpression of CHK1 in THP-1 AML cell line had no impact on LY2603618 sensitivity, shRNA knockdown of CHK1 in the cells resulted in significantly increased sensitivity to the agent. These results suggest that there is a minimum amount of CHK1 that is required for cellular functions and anything above that threshold seems unnecessary.

As expected, LY2603618 treatment of AML cells caused DNA damage, along with decreased Mcl-1 protein levels and apoptosis. Interestingly, our time course experiment demonstrated that induction of DNA damage and decrease of Mcl-1 protein occurred at the same time, which was associated with the initiation of apoptosis. While CHK1 inhibitor-induced DNA damage and cell death is expected, decrease of Mcl-1 is an interesting finding which may be useful for designing combinations with CHK1 inhibitors. As one could expect, this property of LY2603618 rendered its ability to overcome intrinsic resistance to the Bcl-2-selective inhibitor ABT-199, mediated by Mcl-1 in AML cells, leading to synergistic antileukemic interactions between the two agents in both AML cell lines and primary AML patient samples. Although we found that overexpression of Mcl-1 only partially inhibited LY2603618-induced apoptosis, Nijhawan et al. revealed that overexpression of Mcl-1 inhibited UV-induced apoptosis [26]. This difference may be due to the difference in the extent of Mcl-1 overexpression or could suggest that additional factors are also involved in LY2603618-induced apoptosis.

A surprising finding of this study was that LY2603618-induced DNA damage was enhanced by ABT-199 in AML cells, which was potentially responsible for the abolishment of ABT-199-induced increase of Mcl-1. Though the exact mechanism by which ABT-199 treatment enhances DNA damage induced by LY2603618 remains unknown, Bcl-2 has been demonstrated to be associated with numerous DDR proteins, such as APE1, PARP1, Ku70 and BRCA1 (reviewed in [31]), therefore it is plausible that ABT-199 treatment inhibits or enhances Bcl-2′s role in the DDR which can then increase CHK1 inhibitor-induced DNA damage. Studies are underway investigating the molecular mechanism responsible for the enhancement of ABT-199 on LY2603618-induced DNA damage. However, this is not in the scope of this paper.

The CDK inhibitor Roscovitine partially inhibited LY2603618-induced cell death, decrease of Mcl-1, and DNA damage, suggesting that CDK-independent mechanisms of DNA damage induced by LY2603618 treatment also contributed to cell death. As a central player in the DDR, besides regulating the cell cycle checkpoints, CHK1 also plays important roles in DNA repair and stabilization of DNA replication forks [32, 33]. Thus, targeting CHK1 with LY2603618 could inhibit CHK1's involvement in these biological processes, leading to DNA damage independent of CDK activity.

**Figure 7:**
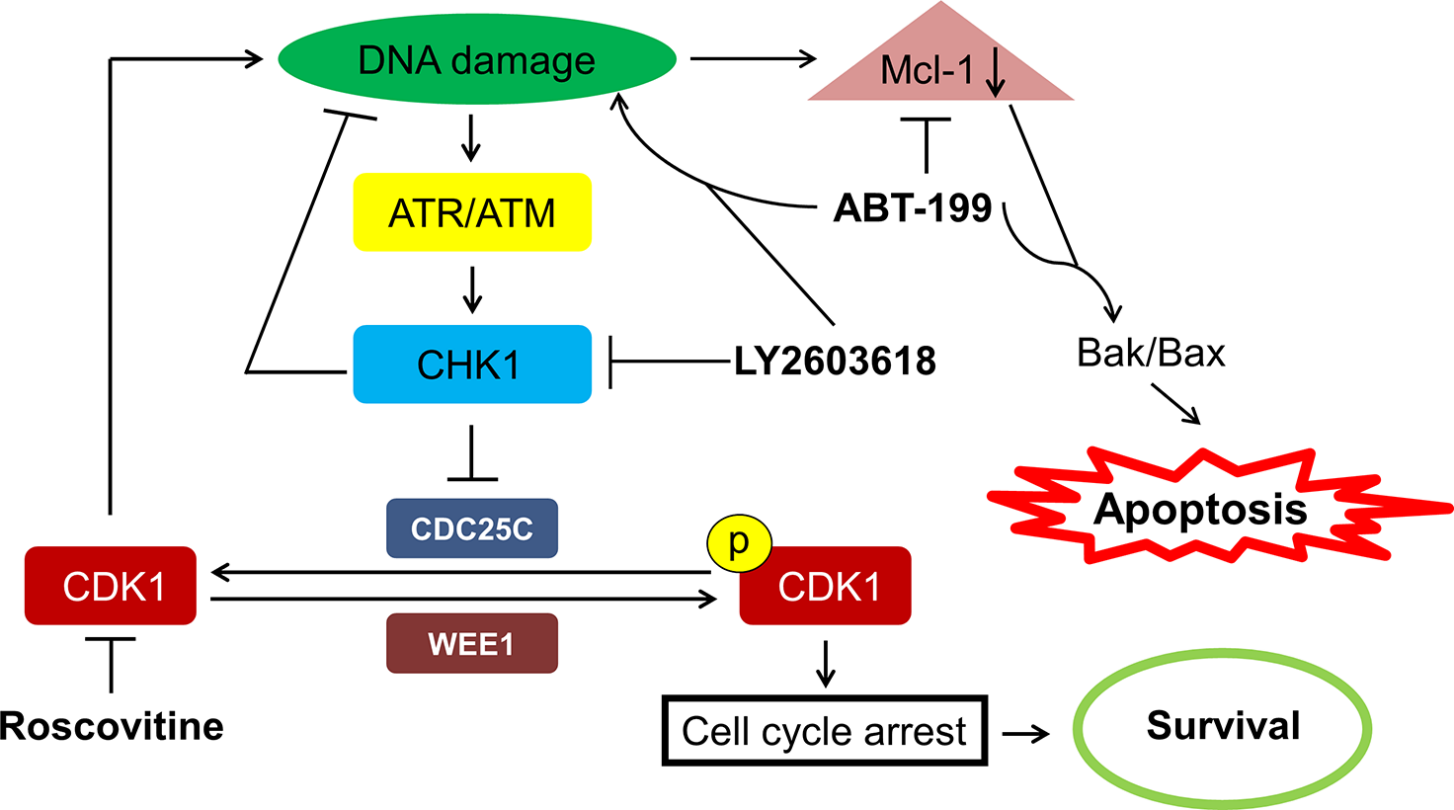
Proposed mechanism of action for LY2603618 alone or in combination with ABT-199 in AML cells LY2603618 treatment inhibits CHK1, which results in CDK-dependent and CDK-independent DNA damage. DNA damage leads to a decrease of Mcl-1 protein levels and apoptosis. ABT-199 treatment in resistant cells leads to increased levels of Mcl-1 protein which can be abrogated by combination with LY2603618 due to enhanced DNA damage induced by the combination of LY2603618 and ABT-199, resulting in synergistic induction of apoptosis.

Taken together, our results as well as those previously reported by others, provide insight into the mechanism of action for the synergistic antileukemic activity of LY2603618 and ABT-199. Our proposed mechanism is presented in Figure 7. LY2603618 treatment inhibits CHK1 resulting in accumulation of DNA damage. DNA damage decreases Mcl-1 protein levels, which decreases its inhibitory effect on pro-apoptotic proteins, ultimately resulting in the initiation of apoptosis. In ABT-199 resistant AML cells, ABT-199 treatment results in increased Mcl-1 protein levels. However, when combined with LY2603618, enhanced DNA damage occurs and results in abolishment of the increase of Mcl-1 protein, leading to synergistic induction of apoptosis. In summary, LY2603618 enhances ABT-199 treatment in AML cells. The toxicities in patients associated with either drug have been reported [27, 34–37], and do not appear to overlap. Further, the toxicities that are associated with the individual treatments are manageable; suggesting that combination therapy associated toxicities would be manageable. Although our study used a limited number of patient samples, and therefore may not necessarily represent the full spectrum of AML, our data supports the further development of CHK1 inhibition in combination with ABT-199.

## MATERIALS AND METHODS

### Drugs

LY2603618, Roscovitine, and ABT-199 were purchased from Selleck Chemicals (Houston, TX).

### Cell culture

U937, THP-1, MV4-11, and HL60 cell lines were purchased from the American Type Culture Collection (Manassas, VA). The CMY, CMS, and CTS cell lines were gifts from Dr. A Fuse from the National Institute of Infectious Diseases, Tokyo, Japan. The CMK, NB4, and OCI-AML3 cell lines were purchased from the German Collection of Microorganisms and Cell Cultures (DSMZ, Braunschweig, Germany). MOLM-13 cells were purchased from AddexBio (San Diego, CA). The cell lines were cultured in RPMI 1640 (except OCI-AML3, which was cultured in alpha-MEM) with 10–15% fetal bovine serum (Life Technologies, Grand Island, NY), 2 mM L-glutamine, 100 U/ml penicillin and 100 μg/ml streptomycin. All cells were cultured in a 37°C humidified atmosphere containing 5% CO2/95% air.

Diagnostic AML blast samples derived from patients either at initial diagnosis or at relapse were purified by standard Ficoll-Hypaque density centrifugation, then cultured in RPMI 1640 with 20% fetal bovine serum supplemented with ITS solution (Sigma-Aldrich, St. Louis, MO, USA) and 20% supernatant of the 5637 bladder cancer cell line (as a source of granulocyte-macrophage colony-stimulating factor [12, 38, 39]).

### Clinical samples

Diagnostic AML blast samples were obtained from the First Hospital of Jilin University. Written informed consent was provided according to the Declaration of Helsinki. This study was approved and carried out in accordance with the guidelines set forth by the Human Ethics Committee of the First Hospital of Jilin University. Clinical samples were screened for gene mutations by PCR amplification and automated DNA sequencing and for fusion genes by real-time RT-PCR, as described previously [12, 40].

### *In vitro* cytotoxicity assays

*In vitro* cytotoxicities of the AML cells were measured by using MTT (3-[4, 5-dimethyl-thiazol-2-yl]-2, 5-diphenyltetrazoliumbromide, Sigma-Aldrich), as previously described [41, 42]. Briefly, the cells were treated with variable concentrations of LY2603618, ABT-199, or in combination for 72 hours. MTT was added to a final concentration of 1 mM and cells were incubated for 4 hours at 37^°^C. The cells were lysed overnight using 10% SDS in 10 mM HCl and plates were read at 590 nm using a microplate reader. IC_50_ values were calculated as drug concentrations necessary to inhibit 50% growth compared to vehicle control treated cells. The IC_50_ values for the cell lines are presented as mean values ± standard errors from at least three independent experiments. The IC_50_ values for the patient samples are means of duplicates from one experiment, due to limited sample. Patient samples for the combined drug treatments were chosen solely based on sample availability.

### Quantification of gene expression by real-time RT-PCR

Total RNA was extracted using TRIzol (Life Technologies) and cDNAs were prepared from 2 μg total RNA using random hexamer primers and a RT-PCR kit (Life Technologies), and purified using the QIAquick PCR Purification Kit (Qiagen, Valencia, CA) as previously described [12, 42, 43]. *CHK1* (Hs00967506_m1) transcripts were quantitated using TaqMan probes (Life Technologies) and a LightCycler^®^ 480 real-time PCR machine (Roche Diagnostics, Indianapolis, IN), based on the manufacturer's instructions. Real-time PCR data are presented as means of duplicates from one experiment, due to limited sample, and results were normalized to *GAPDH* (4333764-1007034) transcripts. Fold changes were calculated using the comparative *C_t_* method [44].

### Western blot analysis

Cells were lysed in the presence of protease and phosphatase inhibitors (Roche Diagnostics). Whole cell lysates were subjected to SDS-polyacrylamide gel electrophoresis, electrophoretically transferred onto polyvinylidene difluoride (PVDF) membranes (Thermo Fisher Inc., Rockford, IL) and immunoblotted with anti-p-CDK1 (Y15) (9111), -CDK1 (9112), -p-CDC25C (S216) (9528), -PARP (9542), -Bax (2774), -Bak (3814), -p-H3 (S10) (9701), -cleaved caspase-3 (9661), -Bcl-2 (2876), -Bcl-xL (2764), -Mcl-1 (4572), -Bim (2819), -CHK1 (2345), -p-CHK1 (S345) (2341), -γH2AX (2577, Cell Signaling Technology, Danvers, MA, USA) or -β-actin antibody (Sigma-Aldrich), as previously described [45, 46]. Immunoreactive proteins were visualized using the Odyssey Infrared Imaging System (Li-Cor, Lincoln, NE, USA), as described by the manufacturer. Western blots were repeated at least three times and one representative blot is shown.

### Apoptosis

AML cells were treated with LY2603618 and Roscovitine, alone or in combination, or with LY and ABT-199, alone or in combination, and subjected to flow cytometry analysis to determine drug-induced apoptosis using an Annexin V-fluorescein isothiocyanate (FITC)/propidium iodide (PI) apoptosis Kit (Beckman Coulter; Brea, CA, USA), as previously described [41, 43]. Apoptotic events are presented as percentage of AnnexinV+/PI- and Annexin V+/PI+ ± s.e.m. Experiments with AML cell lines were performed 3 independent times in triplicates, while patient sample experiments were performed once in triplicate due to limited sample. Data are presented as mean ± standard errors from one representative experiment. Patient samples were chosen based on availability of adequate sample for the assay. The extent and direction of antileukemic interaction were determined by calculating the combination index (CI) values using CompuSyn software (Combosyn Inc., Paramus, NJ). CI < 1, CI = 1, and CI > 1 indicate synergistic, additive, and antagonistic effects, respectively [41, 47].

### Cell cycle progression

Cells were treated with the indicated drugs for up to 48 h. The cells were harvested and fixed with ice-cold 80% (v/v) ethanol for 24 h. The cells were pelleted, washed with PBS, and resuspended in PBS containing 50 μg/mL PI, 0.1% Triton X-100 (v/v), and 1 μg/mL DNase-free RNase. DNA content was determined by flow cytometry analysis using a FACS Calibur flow cytometer (Becton Dickinson), as previously described [48]. Cell cycle analysis was performed using ModFit LT 3.0 (Becton Dickinson). Histograms were created using FlowJo v7.6.5 (Tree Star, Ashland, OR, USA).

### Production of lentivirus particles and transduction of AML cells

The pMD-VSV-G and delta 8.2 plasmids were gifts from Dr. Dong at Tulane University. Bak, Bax, CHK1, and non-target control (NTC) shRNA lentiviral vectors were purchased from Sigma-Aldrich. Red fluorescent protein (RFP), CHK1, and Mcl-1 cDNA constructs were purchased from Thermo Fisher Scientific Biosciences (Lafayette, CO). Lentivirus production and transduction were carried out as previously described [28]. Briefly, TLA-HEK293T cells were transfected with pMD-VSV-G, delta 8.2, and lentiviral shRNA constructs using Lipofectamine and Plus reagents (Life Technologies) according to the manufacturer's instructions. Virus containing culture medium was harvested 48 h post-transfection. Cells were transduced overnight using 1 mL of virus supernatant and 4 μg of polybrene and then cultured for an additional 48 h prior to selection with puromycin.

### Alkaline comet assay

U937 cells were treated with the indicated drugs for 8 or 16 h and then subjected to alkaline comet assay, as previously described [28]. Slides were stained with SYBR Gold (Life Technologies), and then imaged on an Olympus BX-40 microscope equipped with a DP72 microscope camera and Olympus cellSens Dimension software (Olympus America Inc., Center Valley, PA). Approximately 50 comets per gel were scored using CometScore (TriTek Corp, Sumerduck, VA). The median percent DNA in the tail was calculated and graphed ± s.e.m.

### Statistical analysis

Differences in cell apoptosis between treated (individually or combined) and untreated cells were compared using the pair-wise two-sample *t*-test. The *p* value for the differences between LY IC_50_s for the groups of patient samples was calculated using the Mann-Whitney two-sample *U* test. The nonparametric Spearman rank correlation coefficient was used to analyze the relationship between LY IC_50_s and CHK1 transcript levels in the primary AML patient samples. Statistical analyses were performed with GraphPad Prism 5.0. Error bars represent ± s.e.m. The level of significance was set at *p* < 0.05.

## SUPPLEMENTARY MATERIAL FIGURES


