# Supplementary material for: Inhibition of CHK1 enhances cell death induced by the Bcl-2-selective inhibitor ABT-199 in acute myeloid leukemia cells
Source: Oncotarget. 2016 May 5;7(23):34785–99. doi: 10.18632/oncotarget.9185 (PMC5085189; doi:10.18632/oncotarget.9185)
Supplement: Supplementary file 1 [file oncotarget-07-34785-s001.pdf]

Inhibition of CHK1 enhances cell death induced by the Bcl-2-selective inhibitor ABT-199 in acute myeloid leukemia cells

Supplementary Materials

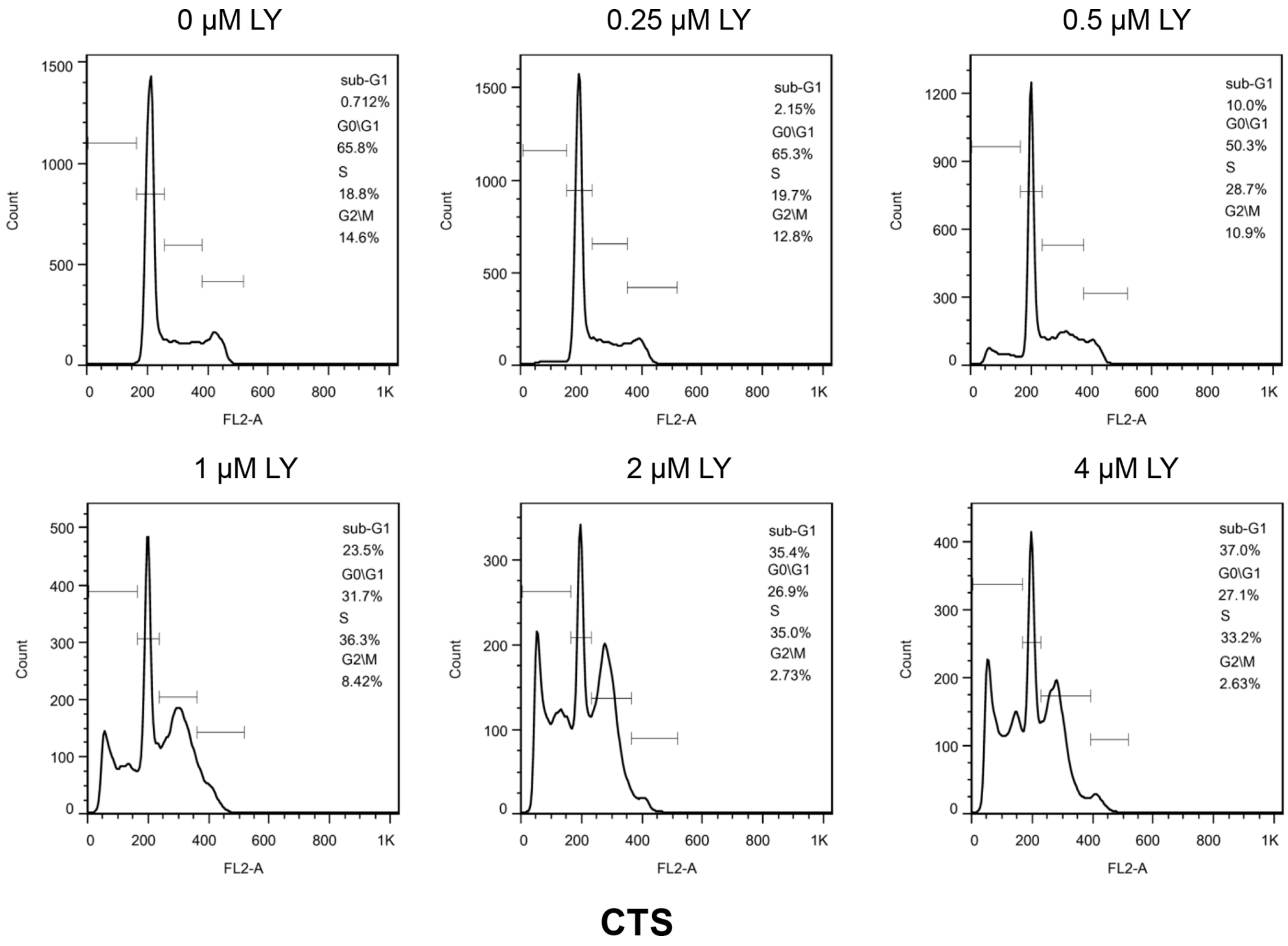

Supplementary Figure S1: CTS cells were treated for 24 h with LY2630618 (LY), then fixed with ethanol and stained with PI for cell cycle analysis.

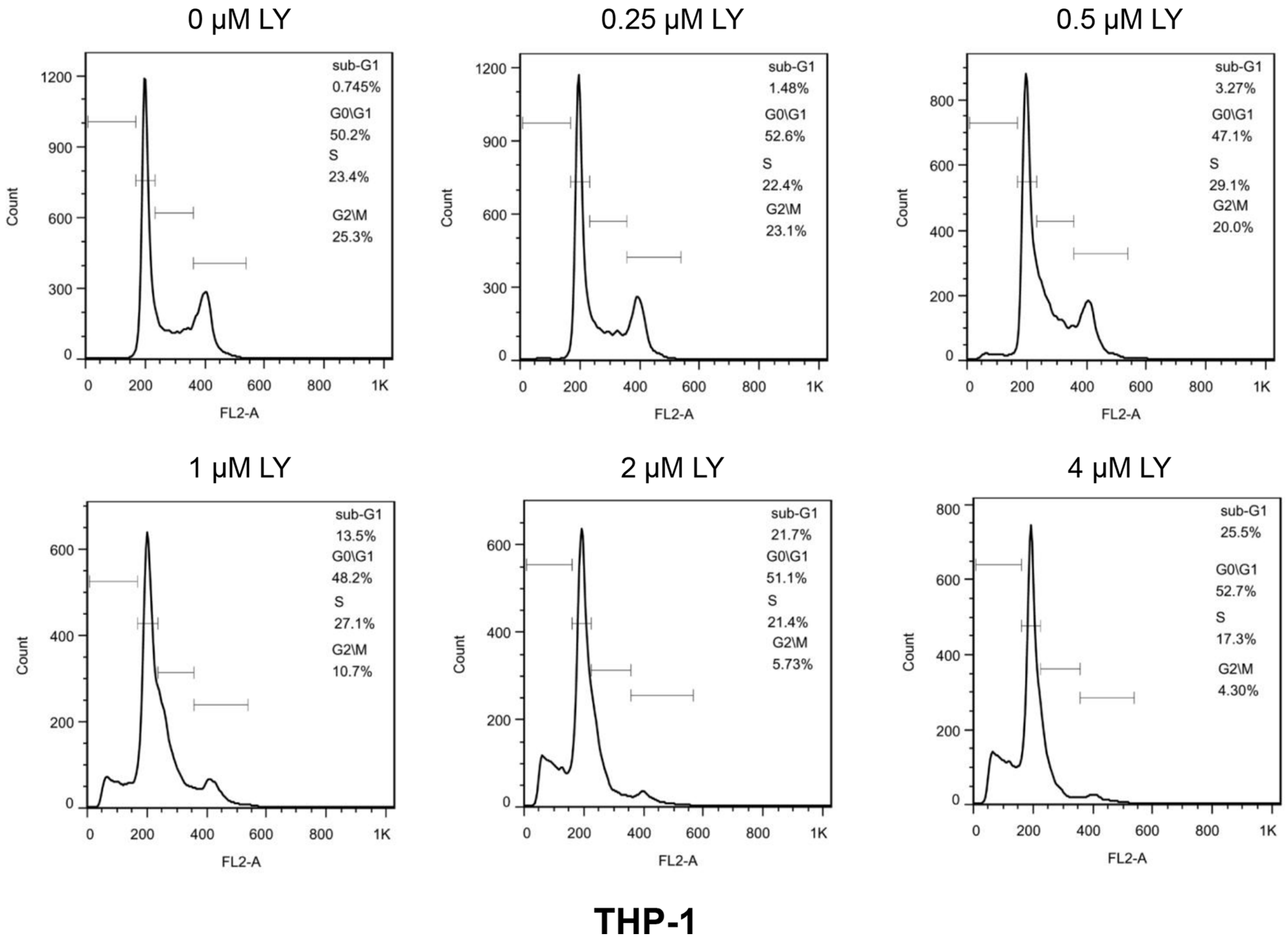

Supplementary Figure S2: THP-1 cells were treated for 24 h with LY, then fixed with ethanol and stained with PI for cell cycle analysis.

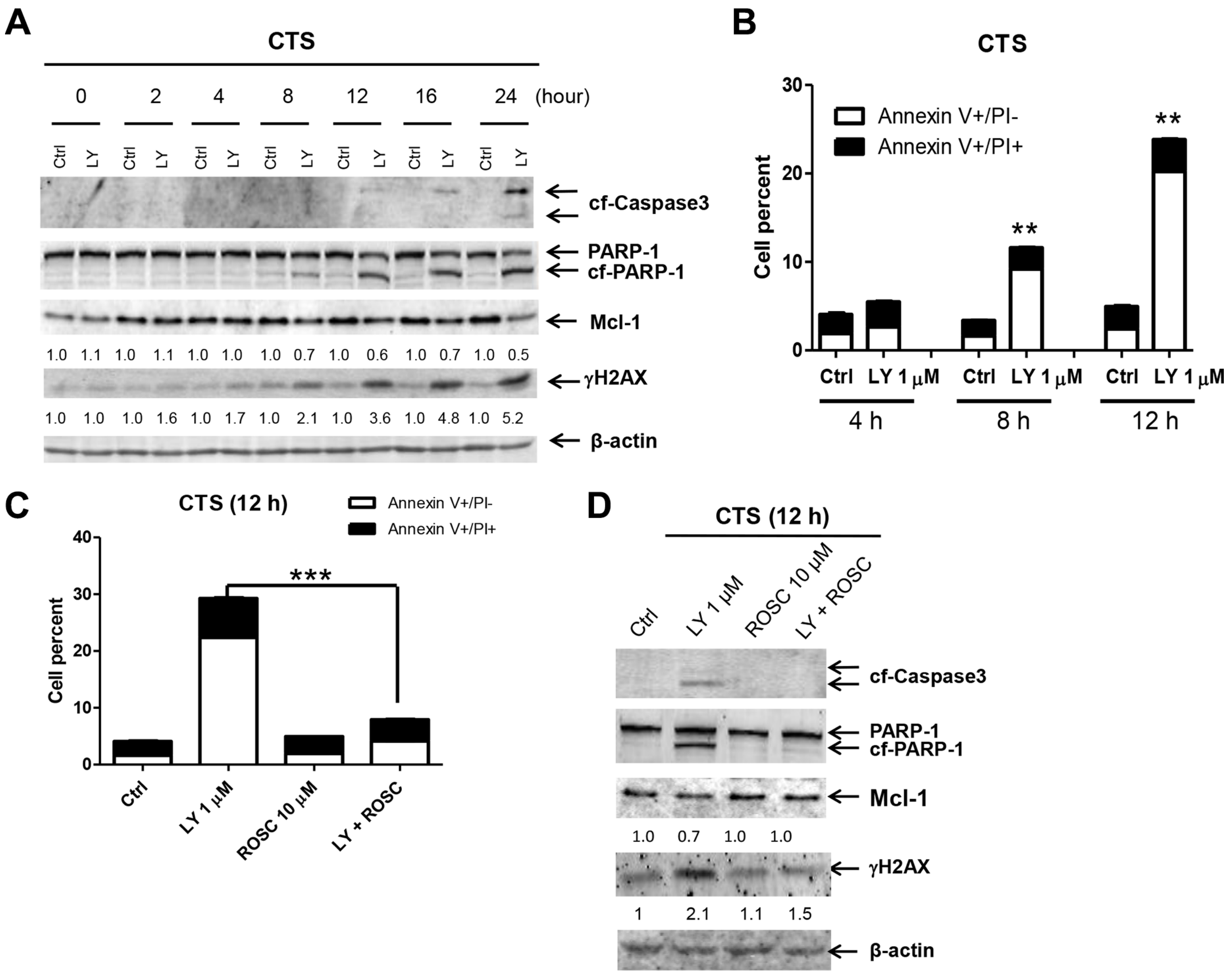

Supplementary Figure S3: (**A**) CTS cells were treated with 4 μM LY or without (control, designated ctrl) for the indicated times. Whole cell lysates were subjected to Western blotting. (**B**) Cells were treated with LY for 4 h, 8 h, and 12 h and then subjected to Annexin V/PI staining and flow cytometry analysis. **indicates $p < 0.005$. (**C**) CTS cells were treated with LY and Roscovitine (ROSC) for 12 h. Cells were then subjected to Annexin V/PI staining and flow cytometry analysis. ***indicates $p < 0.0005$. (**D**) Whole cell lysates were subjected to Western blotting.
